# Endobiliary radiofrequency ablation to remove embedded metal stents

**DOI:** 10.1055/a-2820-4664

**Published:** 2026-03-30

**Authors:** Thomas Lambin, Sarah Leblanc, Bertrand Napoleon

**Affiliations:** 1 89686 Endoscopy Unit, Hôpital Privé Jean Mermoz, Ramsay Santé, Lyon, France

Removal of embedded biliary metal stents is challenging when prolonged indwelling leads to tissue ingrowth. Standard options such as the stent-in-stent technique may fail 1 2 . We report a seven-patient case series in which endobiliary radiofrequency ablation (RFA) combined with the stent-in-stent technique was used to facilitate stent removal ( Video 1 ).

Endobiliary radiofrequency ablation applied to tissue ingrowth within an embedded metal stent, followed by stent-in-stent placement and successful extraction 1 month later.Video 1

All seven patients were men with a mean age of 63.6 ± 14.4 years. The final diagnoses were cholangiocarcinoma ( *n* = 4), chronic calcified pancreatitis ( *n* = 1), autoimmune cholangitis ( *n* = 1), and lymphoma ( *n* = 1). Four patients had a single embedded stent: a fully covered metal stent (FCSEMS) in three patients and an uncovered metal stent (UCSEMS) in one patient. Three patients had two embedded stents (stent-in-stent): two UCSEMS, *n* = 2, and one FCSEMS inside an UCSEMS, *n* = 1. In three patients, previous attempts using the standard stent-in-stent technique had failed. The mean duration between stent placement and RFA was 36 ± 23.2 months (range: 8–72).

**Fig. 1:**
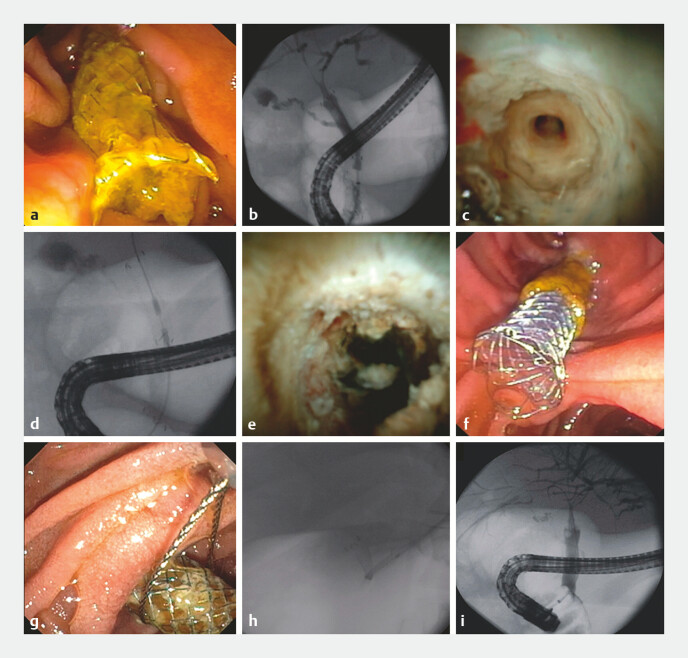
The embedded stent observed by **a** endoscopy and **b** cholangiography. **c** Cholangioscopy showing the stent mesh deeply embedded in the bile duct. **d** Biliary radiofrequency ablation sessions along the embedded areas. **e** Appearance by cholangioscopy after radiofrequency ablation. **f** Placement of a fully covered metal stent within the stent to be removed. **g** Removal of the stents using a snare. **h** Appearance by cholangiography during stent removal. **i** Appearance by cholangiography after the removal of the stent with its visible imprint.

During the procedure, the embedded stent was cannulated, and tissue ingrowth was confirmed by cholangiography. RFA was then applied circumferentially along the ingrowth zones using a 7-Fr catheter at 10 W with a maximum heat of 85°C with single treatment at each location. The size of the probe and the duration of treatment were adapted to the extension of tissue ingrowth observed by cholangiography. The mean (SD) duration of the RFA sessions was 6.5 ± 4.5 min. A FCSEMS was systematically inserted after RFA to promote the necrosis of ingrowth tissue. One month later, stent extraction was attempted using a snare ( Fig. 1 ) rather than forceps to minimize irreversible stent damage. Successful removal occurred in five cases (71.4%), including two previous removal failures with the stent-in-stent technique. Five patients (71.4%) had one session of RFA and two patients (28.6%) had two sessions. Two patients experienced adverse events: a cholecystitis and a biliary peritonitis in one patient and recurrence of cholestasis after successful extraction in another.

This case series suggests that endobiliary RFA may serve as a useful adjunct to facilitate the extraction of long-standing embedded metal stents when the stent-in-stent technique fails. Since the two procedural failures involved long stents (10 cm) in the right hepatic duct, we would not recommend this technique in case of the intrahepatic involvement of the stent. Additional data are required to better define the risk factors associated with procedural failures and to further assess safety considerations.

Endoscopy_UCTN_Code_TTT_1AR_2AZ Endoscopy_UCTN_Code_TTT_1AR_2AG
